# Pyrolysis of Methyl Ricinoleate: Distribution and Characteristics of Fast and Slow Pyrolysis Products

**DOI:** 10.3390/ma15041565

**Published:** 2022-02-19

**Authors:** Xiaoning Mao, Qinglong Xie, Ying Duan, Shangzhi Yu, Yong Nie

**Affiliations:** Biodiesel Engineering Lab of China Petroleum & Chemical Industry Federation, Zhejiang Province Key Lab of Biofuel, Zhejiang University of Technology, Hangzhou 310014, China; 1112001035@zjut.edu.cn (X.M.); xieql@zjut.edu.cn (Q.X.); duanying@zjut.edu.cn (Y.D.); ysz@zjut.edu.cn (S.Y.)

**Keywords:** methyl ricinoleate, undecylenic acid methyl ester, heptanal, fast pyrolysis reaction process, Py-GC/MS, DFT

## Abstract

A stable temperature site and the speed of heating the feedstocks play a key role in pyrolysis processes. In this study, the product distribution arising from pyrolysis of methyl ricinoleate (MR) at 550 °C with low and high heating rates was first studied by pyrolysis–gas chromatography/mass spectrometry (Py-GC/MS). The results show that fast pyrolysis of MR favored the production of undecylenic acid methyl ester (UAME) and heptanal (HEP). Density functional theory (DFT) calculations were employed to reveal the UAME and HEP formation process from pyrolysis of MR. The bond dissociation energies (BDEs) of C–C bonds in MR showed that the C11–C12 bond is the weakest. This suggests that UAME and HEP are two major products. The process of slow and fast MR pyrolysis was the dehydration-first and the pyrolysis-first trend, respectively. The calculated activation energies of MR pyrolysis to UAME and HEP and MR dehydration to 9,12-octadecadienoic acid methyl ester were 287.72 and 238.29 kJ/mol, respectively. The much higher product yields obtained in the fast pyrolysis reactors than those from conventional tubular reactors confirmed the proposed process.

## 1. Introduction

The availability of biomass has great potential for economic benefits, and pyrolysis or catalytic pyrolysis is an economically and feasible technology for the utilization of biomass [1,2,3]. Undecylenic acid methyl ester (UAME) and heptanal (HEP), pyrolysis products of methyl ricinoleate (MR), are considered renewable resources for the chemical industry [4,5]. UAME, owing to its bifunctional nature, can be used in the production of engineering plastics, e.g., Nylon 11 [6,7,8]. HEP is a chemical intermediate to produce fragrance and flavor. Therefore, it is critical to obtain high yields of UAME and HEP from the MR pyrolysis process.

A stable and uniform temperature field and rapid heating of MR play a key role in pyrolysis processes, which determine the yields of UAME and HEP. For the conventional tubular reactors, heat is primarily transferred through conduction, and a temperature gradient usually exists within the reactor, which would cause deep pyrolysis and hence a reduction in UAME and HEP yields [9,10]. Our group proposed microwave and inductive heating coupled with atomization feeding reactors for MR pyrolysis [11,12,13]. The spray heat transfer resulted in rapid heating of the MR feedstock and hence an increase in product yields. However, few studies on the detailed reaction process for MR pyrolysis can be found.

Pyrolysis–gas chromatography/mass spectrometry (Py-GC/MS) combined with density functional theory (DFT) is an advanced research method for the study of pyrolysis products’ distribution. This method has been adopted to study the pyrolysis process of invasive plants and high-density polyethylene [14], holocellulose-based monosaccharides [15] and lignin [16]. Mishra [17] researched the physicochemical characterization, kinetics and thermal degradation behaviors of waste switchgrass pyrolysis products through Py-GC/MS. Wang et al. [18] proposed four possible reaction pathways for cellulose pyrolysis based on Py-GC/MS results. The activation energy of different reaction pathways was then calculated by DFT. Adnan et al. [19] characterized polymethylmethacrylate (PMMA) composites with an inorganic salt of aluminum triiodide (AlI_3_) through Py-GC/MS. The analysis results deduced that the PMMA composite produced less toxic and environmentally friendly substances by the influence of AlI_3_.

The objective of this work was to study the reaction process of MR pyrolysis by Py-GC/MS experiments combined with DFT calculation. The product distribution from slow and fast pyrolysis of MR was analyzed through Py-GC/MS. The bond dissociation energies (BDEs) of C–C bonds in MR, UAME and HEP were calculated by DFT. Moreover, the activation energies of the main reactions postulated based on the Py-GC/MS results were determined by DFT. Furthermore, the proposed reaction trend was confirmed by the experimental results obtained from slow and fast pyrolysis reactors.

## 2. Materials and Methods

### 2.1. Materials

MR was firstly prepared by transesterification of castor oil with methanol over potassium hydroxide as the catalyst. This was followed by purification of MR with a purity of 97% by distillation at the laboratory scale. The main constituents of the other 3% were ricinoleic acid (approximately 2%), methyl stearate and methyl oleate. Castor oil was purchased from Jiangsu Wuxi Haishuo Biological Co. Ltd., Wuxi, China. Methanol (≥99.5%) and potassium hydroxide (≥85%) were obtained from Shanghai Titan Technology Co., Ltd., Shanghai, China.

### 2.2. Py-GC/MS Experiments

The experiments of MR pyrolysis were carried out in a micro-pyrolyzer (EGA/PY-3030D, Frontier Laboratories, Koriyama, Japan), with the volatiles analyzed by GC/MS (GCMS-QP 2010 SE, Shimadzu, Japan). For a specific test, a deactivated stainless-steel sample cup was loaded with about 1 mg of MR. For fast pyrolysis, the loaded cups fell freely into the preheated furnace by gravity in a very short time period, during which the sample was heated to the pyrolysis temperature, ensuring fast pyrolysis. The pyrolysis volatiles were directly swept into the GC/MS for analysis using helium as the carrier gas. For slow pyrolysis, the temperature increased from 200 to 550 °C at two different heating rates which were 5 °C/min and 20 °C/min. The pyrolysis volatiles were condensed in the chromatographic column head and subsequently swept into the GC/MS after the temperature program.

The chromatographic separation and identification of pyrolysis products were performed using a mass spectrometer equipped with a capillary column (Rtx-5MS, 30 m × 0.25 mm × 0.25 μm). The GC oven temperature program began with 40 °C, held for 2 min, increased to 320 °C at 20 °C/min and finally held at 320 °C for 13 min.

### 2.3. DFT Computational Details

All the geometry optimizations and energy calculations in the DFT study were performed using the Gaussian 09 (Gaussian Inc., Wallingford, CT, USA) suite of programs with the B3LYP/Def2-TZVP basis set. When the optimization results of reactants and products had no imaginary frequencies, while transition states (TS) had sole imaginary frequencies, it was considered that the geometry was feasible. Activation energies for reactions were estimated from the relative energies between the transition state and the reactant.

## 3. Results and Discussion

### 3.1. Product Distribution of MR Pyrolysis at Different Heating Rates

**Figure 1:**
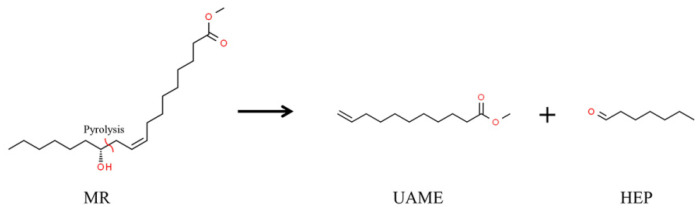
The chemical reaction of MR pyrolysis to UAME and HEP.

The main product distribution from pyrolysis of MR at different heating rates to 550 °C is shown in Table 1. The area percentage of MR was 56.95% and 42.30% for slow pyrolysis, much higher than that of 20.23% for fast pyrolysis. Thus, the MR conversion was improved at a higher heating rate. The area percentages of non-pyrolysis products, i.e., dehydration products and unreacted MR, were 73.79% and 56.02% at the heating rates of 5 °C/min and 20 °C/min, respectively. The value was significantly decreased to 32.76% for fast pyrolysis. This indicated that MR showed a dehydration and evaporation trend during slow pyrolysis. Overall, the process of slow and fast pyrolysis of MR was the dehydration-first and the pyrolysis-first trend, respectively. In addition, by pyrolysis of 1 mol of MR, it is possible, in theory, to obtain 1 mol of HEP and 1 mol of UAME, with the chemical reaction shown in Figure 1. However, the molar ratio of HEP and UAME in pyrolysis products was not 1:1. This was probably due to the purity of the feedstock not being 100%, where there is ricinoleic acid in the raw feedstock used for pyrolysis [20].

As listed in Table 1, the most prominent products in fast pyrolysis were UAME and HEP, with the area percentages being 42.21% and 16.21%, respectively. The values for these two target products in MR pyrolysis were obviously higher than those obtained at the heating rates of 5 °C/min and 20 °C/min. This was primarily due to the temperature distribution of MR being uniform, and the energy being rapidly transferred to MR for pyrolysis [21]. Rapid heating of MR contributes to transferring the pyrolysis volatiles from the high-temperature site, and avoiding the second pyrolysis. In addition, the residence time of MR was extended with slow pyrolysis, resulting in distinct pyrolysis stages and more secondary pyrolysis of products [22,23].

### 3.2. Product Distribution of Fast MR Pyrolysis at Different Temperatures

The product distribution of fast MR pyrolysis at different temperatures is shown in Table 2. The MR conversion increased with the increasing temperature from 400 to 600 °C. The relative area percentages of pyrolysis products were 2.86%, 28.8% and 95.51% at the pyrolysis temperatures of 400 °C, 500 °C and 600 °C, respectively. The significant increase in the number of pyrolysis products at higher temperatures was mainly due to the increasing cleavage of the C–C bond of MR. In addition, smaller molecules, e.g., ethanol, 1,3-butadien, 2-pentene, propenoic acid methyl ester and pentenoic acid methyl ester, were detected at 600 °C. This indicated that secondary thermal cracking of UAME and HEP was enhanced at higher temperatures.

### 3.3. DFT Studies on MR Pyrolysis

**Figure 2:**
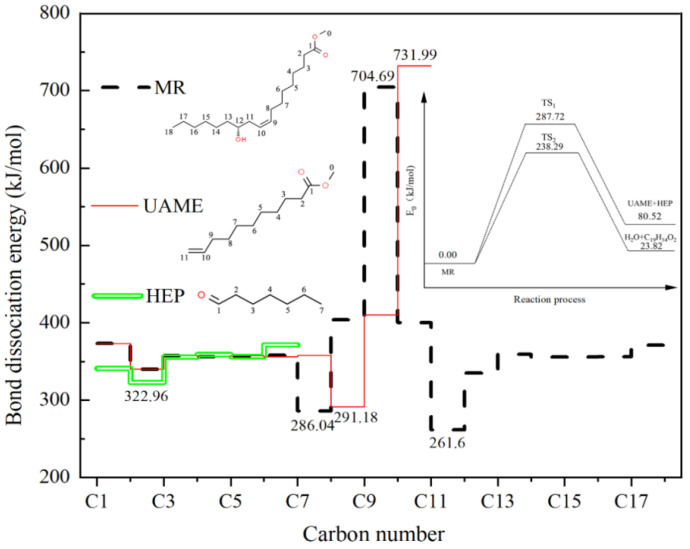
The C–C bond dissociation energy and potential energy profile in main products and reaction.

Based on the Py-GC/MS results of MR pyrolysis, the dehydration-first trend and the pyrolysis-first trend were proposed. To verify the trend, DFT studies were carried out to analyze the BDEs and reaction pathways. The bond dissociation order of C–C bonds in MR, UAME and HEP during the pyrolysis process can be predicted by the BDEs. The results of BDEs and the calculated activation energy for different reaction pathways are shown in Figure 2. The BDE of the C11–C12 bond is the lowest in MR, and the BDE of the C7–C8 bond is slightly higher than that of the C11–C12 bond. This suggests that UAME and HEP are two major products during MR pyrolysis, with heptenoic acid methyl ester and 1,3-butadiene as the by-products. The BDE results are in agreement with the experimental results of MR pyrolysis. In addition, Botton et al. [22] reported that undesired products can be produced through the secondary pyrolysis of MR, forming methyl ester with 7–11 carbons in the chain, and also the presence of other methyl esters such as methyl palmitate, methyl stearate, methyl oleate, methyl linoleate and methyl linolenate. This is also in agreement with the calculated BDE results. The calculated activation energy of MR pyrolysis to UAME and HEP was 287.27 kJ/mol, and the calculated activation energy of MR dehydration to 9,12-octadecadienoic acid methyl ester was 238.29 kJ/mol. Both MR pyrolysis and dehydration belong to endothermic reactions, with the reaction heat being 80.52 and 23.82 kJ/mol, respectively. Thus, MR dehydration could occur at lower temperatures, yet the pyrolysis reaction would be more enhanced at higher temperatures. Fast pyrolysis favored the selectivity of UAME and HEP.

**Figure 3:**
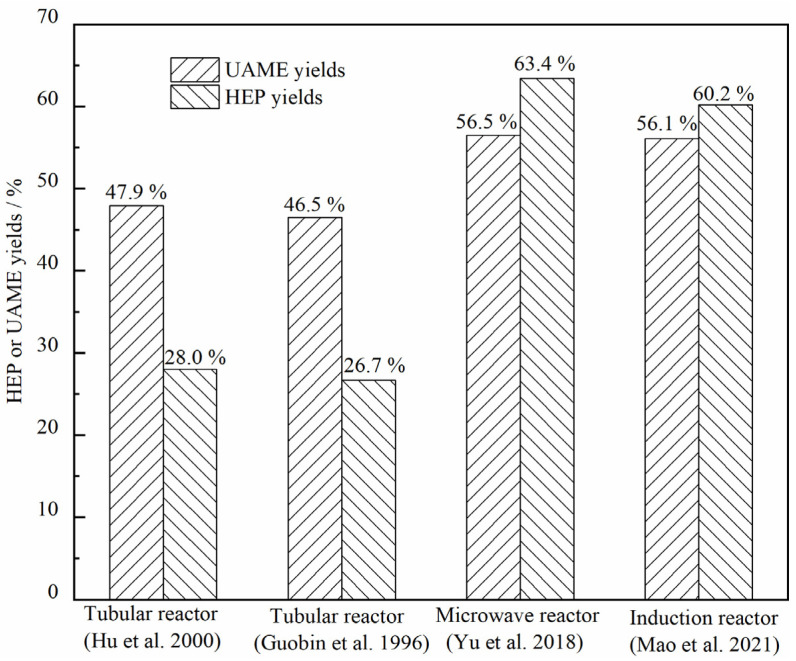
Comparison of different types of reactors for MR pyrolysis [9,10,11,13].

The reactors reported for MR pyrolysis include tubular reactors [9,10], microwave heated reactors [13] and inductively heated reactors [11]. Among these, microwave heated and inductively heated reactors were adopted to achieve fast pyrolysis of MR with atomization feeding. The comparison of UAME and HEP yields obtained from different reactors is displayed in Figure 3. The product yields for the fast pyrolysis reactors were obviously higher than those from the conventional tubular reactors. Therefore, the fast pyrolysis favored MR pyrolysis to UAME and HEP, which verified the proposed reaction trend from the Py-GC/MS coupled with DFT studies.

## 4. Conclusions

The product distribution from pyrolysis of MR with low and high heating rates was investigated by Py-GC/MS for the first time. The yields of UAME and HEP with high heating rates were obviously higher than those obtained with low heating rates. The MR conversion increased with increasing pyrolysis temperature. The results of the DFT studies are in agreement with the experimental results. The process for slow and fast MR pyrolysis was the dehydration-first and the pyrolysis-first trend, respectively. However, the kinetics of MR pyrolysis should be studied for further insights. Overall, the deduced process could provide insight into the MR pyrolysis behavior and the design of pyrolysis reactors.

## Figures and Tables

**Table 1:** Main products’ distribution obtained from pyrolysis of MR with different heating rates to 550 °C (area percentage).

Compound	Formula	Area Percentage/%		
		5 °C/min	20 °C/min	Fast Pyrolysis
2-Ethyl-1-butanol	C_6_H_14_O	1.29	1.44	—
HEP	C_7_H_14_O	—	2.63	16.12
1-Heptanoic acid	C_7_H_14_O_2_	1.72	3.35	—
2-Octanone	C_8_H_16_O	—	0.64	—
(2S)-2-Octanol	C_8_H_18_O	—	0.54	—
6-Heptenoic acid methyl ester	C_8_H_14_O_2_	0.24	0.36	1.7
2-Nonenal, (2E)-	C_9_H_16_O	0.38	1.01	—
2-Octenoic acid, methyl ester, (2E)-	C_9_H_16_O_2_	0.23	0.37	0.99
Caprylic acid methyl ester	C_9_H_18_O_2_	0.66	1.16	—
Monomethyl suberate	C_9_H_16_O_4_	0.45	0.94	—
4-Decanone	C_10_H_20_O	—	0.37	—
Methyl 3-cyclohexylpropanoate	C_10_H_18_O_2_	0.76	1.18	—
Methyl 9-oxononanoate	C_10_H_18_O_3_	2.03	3.03	1.39
Undecynol	C_11_H_20_O	0.19	0.68	—
Dimethyl azelate	C_11_H_20_O_4_	0.69	0.86	—
Decanoic acid methyl ester	C_11_H_22_O_2_	—	1.23	—
UAME	C_12_H_22_O_2_	0.54	0.91	42.21
1-Heptadecene	C_17_H_34_	0.63	—	—
9-Hexadecenoic acid, methyl ester, (9Z)-	C_17_H_32_O_2_	0.96	0.97	—
Methyl 8-(2-hexylcyclopropyl) octanoate	C_18_H_34_O_2_	5.14	5.03	—
9,12-Octadecadienoic acid, methyl ester, (9Z,12Z)-	C_19_H_34_O_2_	3.30	0.90	—
9,15-Octadecadienoic acid, methyl ester, (9E,15E)-	C_19_H_34_O_2_	0.47	0.85	9.86
6-Octadecenoic acid, methyl ester, (6Z)-	C_19_H_36_O_2_	1.07	1.47	—
6-Octadecenoic acid, methyl ester, (6E)-	C_19_H_36_O_2_	0.83	1.34	—
11-Octadecenoic acid, methyl ester, (11Z)-	C_19_H_36_O_2_	6.37	4.50	2.67
11-Octadecenoic acid, methyl ester, (11E)-	C_19_H_36_O_2_	3.40	2.31	—
9-Octadecenoic acid, methyl ester, (9Z)-	C_19_H_36_O_2_	0.28	0.31	—
9-Octadecenoic acid, methyl ester, (9E)-	C_19_H_36_O_2_	1.12	2.04	—
MR	C_19_H_36_O_3_	56.95	42.30	20.23

**Table 2:** Main products’ distribution obtained from fast pyrolysis of MR with different temperatures (area percentage).

Compound	Formula	Area Percentage/%		
		400 °C	500 °C	600 °C
Ethanol	C_2_H_6_O	—	—	3.43
1,3-Butadiene	C_4_H_6_	—	—	8.24
2-Propenoic acid methyl ester	C_4_H_6_O_2_	—	—	4.75
2-Pentene (Z)-	C_5_H_10_	—	—	4.76
1-Hexene	C_6_H_12_	—	—	4.14
4-Pentenoic acid methyl ester	C_6_H_10_O_2_	—	—	1.10
Benzene	C_6_H_6_	—	—	4.44
1,3-Cyclohexadiene	C_6_H_8_	—	—	2.03
Toluene	C_7_H_8_	—	—	2.59
HEP	C_7_H_14_O	2.25	8.83	9.50
5-Hexenoic acid methyl ester	C_7_H_12_O_2_	—	—	1.81
1-Octene	C_8_H_16_	—	—	1.36
6-Heptenoic acid methyl ester	C_8_H_14_O_2_	—	0.58	3.34
4-Octenoic acid methyl ester (Z)-	C_9_H_16_O_2_	—	—	2.66
Methyl 9-oxononanoate	C_10_H_18_O_3_	—	1.45	—
UAME	C_12_H_22_O_2_	0.61	19.39	41.37
9,12-Octadecadienoic acid, methyl ester, (9E,12E)-	C_19_H_34_O_2_	—	2.36	—
9,11-Octadecadienoic acid, methyl ester, (9Z,11Z)-	C_19_H_34_O_2_	—	2.47	—
9,12-Octadecadienoic acid, methyl ester, (9Z,12Z)-	C_19_H_34_O_2_	—	1.43	—
6-Octadecenoic acid, methyl ester, (6Z)-	C_19_H_36_O_2_	—	2.66	—
9-Octadecenoic acid, methyl ester, (9E)-	C_19_H_36_O_2_	—	0.64	—
MR	C_19_H_36_O_3_	86.40	54.45	—
